# Association between Osteoarthritis and the Triglyceride–Glucose Index in the Korean Population: A Large-Scale Cross-Sectional Study

**DOI:** 10.3390/jcm14207226

**Published:** 2025-10-13

**Authors:** Jeong Hee Chi, Bum Ju Lee

**Affiliations:** 1 Department of Computer Science and Engineering, Konkuk University, Seoul 05029, Republic of Korea; jhchi@konkuk.ac.kr; 2 Digital Health Research Division, Korea Institute of Oriental Medicine, 1672 Yuseong-daero, Yuseong-gu, Daejeon 34054, Republic of Korea

**Keywords:** osteoarthritis, triglyceride, glucose, anthropometry, association

## Abstract

**Background:** Osteoarthritis (OA) is associated with triglyceride–glucose (TyG) index values. However, the evidence supporting this association remains controversial. This study explores the associations of OA with the TyG index and with a combination of the TyG index and obesity in the Korean population. **Methods:** A total of 15,128 subjects (including 6575 men and 8553 women) were included in this analysis. Complex sample binary logistic regression was used to analyze the associations of OA with TyG, TyG-body mass index (TyG-BMI), TyG-waist circumference (TyG-WC), and the TyG-waist-to-height ratio (TyG-WHtR). We applied the Bonferroni correction to account for multiple comparisons. **Results:** The prevalence of OA in the Korean population was 7.3% in men and 24.3% in women. The TyG was not associated with OA. In men, the odds ratio (OR) for the TyG was 0.89 (95% CI, 0.79–1.00; *p* = 0.315) per one standard deviation increase in the crude model, and the adjusted OR was 0.98 (95% CI, 0.86–1.11; adjusted *p* > 0.999) in the adjusted model. In women, the OR for the TyG was 1.27 (95% CI, 1.19–1.35; *p* < 0.001), whereas the adjusted OR was 1.08 (95% CI, 1.01–1.16; adjusted *p* = 0.236). In the adjusted models, TyG-BMI, TyG-WC, and TyG-WHtR were not associated with OA in men but strongly associated with OA in women. **Conclusions:** The TyG index was not associated with OA in the Korean population. The strength of the associations between OA and the TyG indices combined with obesity was lower than the that of the associations between OA and BMI, WC, and WHtR individually.

## 1. Introduction

Osteoarthritis (OA) is a major public health issue in middle-aged and older populations and represents the most prevalent form of arthritis [1,2]. According to the reports of GBD 2021 Osteoarthritis [1], 595 million people had OA in 2020, thus accounting or 7.6% of the global population. OA affects quality of life and daily life activities and is a leading cause of chronic pain, disability, morbidity, and mortality [1,2]. OA most commonly occurs in the appendicular joints of the knees, hips, and hands and is the most common pathological disease characterized by subchondral bone perfusion abnormalities [1,3,4]. Modifiable risk factors for OA include joint injury, obesity or high body mass index (BMI), muscle weakness, smoking, and high-level physical occupations [1,5,6,7], whereas nonmodifiable risk factors include age, sex, hypertension, and diabetes [3,7,8,9]. Clinical management or treatments for OA include physical therapy, exercise and weight loss, pain medication, surgical treatment, nonsteroidal anti-inflammatory drugs, and endothelium-targeted strategies [1,3,4].

Recently, several studies have reported an association between triglyceride–glucose (TyG) index values and various diseases. The TyG index is a predictor of insulin resistance (IR), which has been identified as a potential risk factor for OA, metabolic syndrome, stroke, Alzheimer’s disease, depression, diabetes, hypertension, cardiovascular disease, dementia, and nonalcoholic fatty liver disease [7,10,11,12]. In studies suggesting an association between TyG and OA, insulin resistance has been shown to be closely related to metabolic dysfunction, inflammation, bone mass and density, and obesity [7,13]. Adipose tissue is an important source of cytokines and adipokines, which may contribute to the development and progression of arthritis [12,13,14]. Individuals with obesity tend to have higher serum levels of tumor necrosis factor-alpha (TNF-α) and interleukin-6 (IL-6) [14]. Elevated levels of TNF-α and IL-6 have also been detected in the subchondral bone and cartilage of OA patients [14]. Furthermore, TNF-α promotes the development of insulin resistance and obesity [14]. Therefore, both adipose tissue and insulin resistance represent important biological risk factors for OA [7,12,13,14]. However, although OA has been associated with TyG index values [7,12,13,15,16], the evidence supporting this association remains controversial. Specifically, several studies have reported that the TyG index is strongly associated with OA [7,12,13,15,16], whereas some studies have argued that the TyG index is only weakly associated or not associated with OA [11,17,18]. For example, the study by Huang et al. [7] demonstrated an association between a higher TyG index and an increased risk of OA (odds ratio [OR] = 7.34 [95% confidence interval (CI) = 2.25–23.93], *p* = 0.001) after adjusting for various potential covariates in 3921 participants from the U.S. population. In contrast, Lan et al. [11] reported no significant association between TyG index and OA (OR = 1.23 [95% CI = 0.93–1.62], *p* = 0.151) after adjusting for multiple covariates in 14,715 participants from the adult U.S. population. Furthermore, obesity and IR are major risk factors for OA development [11]. Therefore, several studies have reported that TyG indices combined with obesity are strongly associated with OA [11,17,18,19]. Therefore, this study aimed to examine the association between the TyG index value and OA in the Korean population. Also, we examined the association between OA and TyG indices combined with obesity. Previous studies have mainly focused on the association between OA and the TyG index or TyG indices combined with obesity indices, and the findings remain controversial. In addition, few studies have compared sex differences in the association between OA and TyG index. Therefore, the present study investigated the association of OA with TyG index and TyG indices combined with obesity indices, stratified by sex, through a comprehensive cross-sectional analysis using a large-scale dataset from the Korean population.

## 2. Materials and Methods

### 2.1. Study Design and Target Population

**Figure 1:**
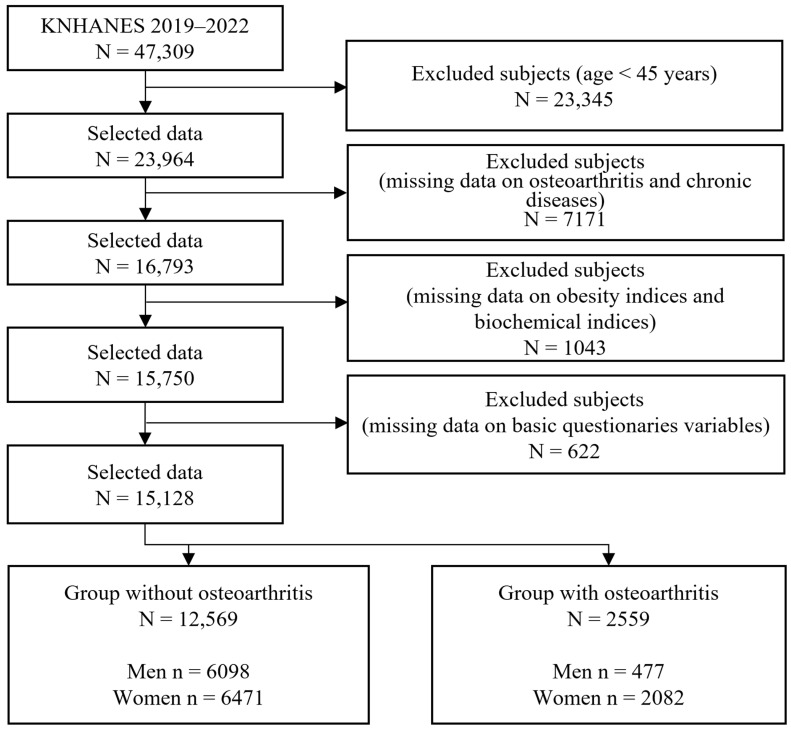
Sample selection criteria and procedure.

In this large-scale cross-sectional study, we used data from the Korea National Health and Nutrition Examination Survey (KNHANES), which focuses on a national representative sample of the Korean population. The KNHANES provides reliable statistics on basic demographic characteristics, nutritional intake status, chronic disease, biochemical profiles, anthropometric measurements and health examinations, and health behavior and status for the Korean population. Additionally, a complex survey sample design that considers weighting, clustering, and stratification was used to address the KNHANES data to ensure that these data were representative of the entire Korean population. Initially, 47,309 subjects participated in the KNHANES from 2014 to 2019. The target population of the present study was adults aged over 45 years because the prevalence of OA in the Korean population aged under 45 years is very low, and high TyG values increase the incidence of arthritis in the population aged over 45 years [12]. Subjects with missing values for major variables, such as biochemical profiles, obesity indices, and important sociodemographic and socioeconomic characteristics, were excluded from the final analysis. Ultimately, a total of 15,128 subjects (6575 men and 8553 women) were included in the study. The specific inclusion and exclusion criteria are presented in Figure 1.

The institutional review board (IRB) of the Korea Disease Control and Prevention Agency (KDCA) approved the KNHANES (IRBs: 2018-01-03-C-A, 2018-01-03-P-A, and 2013-12EXP-03-5C). Furthermore, the IRB of the Korea Institute of Oriental Medicine approved the use of publicly available anonymized KNHANES data (IRB No. I-2501/001-001). All the subjects provided written informed consent before they participated in the KNHANES. Publicly available KNHANES data did not contain any personally identifiable information. The present study was conducted in accordance with the principles of the Declaration of Helsinki.

### 2.2. Definition of Osteoarthritis

In this study, the OA group was defined on the basis of patients who were diagnosed by a doctor and currently suffering from OA. As in previous studies [12,13,17,19], the medical history of doctor-diagnosed OA was obtained by a self-report questionnaire. Additionally, to mitigate respondent recall bias, face-to-face health interviews were conducted with well-trained staff or experts in accordance with the guidelines. The interviewer asked, “Have you been diagnosed with OA by a doctor?” and “Are you currently suffering from OA?” If the respondents answered “Yes” to both questions, they were included in the OA group. Additionally, subjects who were diagnosed with osteoporosis in the past but had since been cured were included in the non-OA group.

### 2.3. Covariates

Based on previous studies reporting associations between OA and TyG, we identified potential confounders that may influence the association. The confounders were as follows: age [7,11,12,13,15,16,17,18,19], education level (tercile) [7,11,12,13,15,16,17,18,19], household income (quartile) [7,11,13,17,18,19], alcohol consumption (yes or no) [7,11,12,17,18,19], smoking (yes or no) [7,11,12,16,17,18,19], weekly walking or exercise (yes or no) [7,13,16], marital status (married or single) [12,13,16,19], and menopause (in women only; yes or no) [20,21]. Diabetes or hypertriglyceridemia status was not used as covariates because the TyG index is derived from triglyceride and glucose levels. A detailed description of these confounders is shown in Table 1.

### 2.4. Measurements and Laboratory Tests

Measurements for obesity indices were performed by specialized staff and experts based on standard protocols. Height and weight measurements of all participants were conducted on the basis of automatic measurement equipment (JENIX DS-102; Dong Sahn Jenix Co., Seoul, Republic of Korea) and measured in units of 0.1 cm and 0.1 kg, respectively. WC was measured with a flexible plastic tape (Seca 200, Hamburg, Germany). The WHtR was calculated by dividing WC by height. BMI was obtained as weight divided by height squared (kg/m^2^).

For biochemical tests, we obtained blood samples from all participants after a minimum fasting period of 8 h based on standard examination protocols. Fasting plasma glucose and triglyceride concentrations were measured by standard enzymatic methods using an automatic analyzer (Hitachi Automatic Analyzer 7600; Hitachi Co., Ltd., Tokyo, Japan). Diastolic and systolic blood pressures (DBP and SBP, respectively) were measured three times using a mercury sphygmomanometer (Baumanometer Wall Unit 33 (0850); Baum Inc., Copiague, NY, USA). The mean of the second and third measurements was used in the analysis. The TyG index was calculated with formulas based on a previous study [22]: TyG = Ln [TG (mg/dL) × glucose (mg/dL)/2)]. Additionally, TyG indices combined with obesity indices were calculated on the basis of a formula developed in accordance with previous studies [11,15,17,19,23]: TyG-BMI = TyG × BMI, TyG-WC = TyG × WC (cm), and TyG-WHtR = TyG × WHtR.

### 2.5. Statistical Analysis

All statistical analyses were conducted using the complex sampling design module in SPSS version 28 (IBM SPSS, Inc., Chicago, IL, USA) to account for the survey design of KNHANES. To produce nationally representative estimates, all analyses incorporated the official survey weights, strata, and cluster variables, in accordance with the KNHANES guidelines. This approach was applied to all results, including the descriptive statistics in Table 1 and the odds ratios derived from the complex sample logistic regression models. Further details on the weighting, stratification, and clustering procedures of the KNHANES data are provided in reference [24]. To examine the statistical significance of sex differences between men and women, *t*-tests with general linear models and Rao–Scott chi-square tests were performed for continuous variables and categorical variables, respectively. The associations of OA with the obesity indices, the TyG index, and the TyG index combined with obesity were examined using complex sample binary logistic regression for both crude and adjusted models after applying standardization. A detailed description and statistical analysis methods of a complex survey sample design are available [24,25]. Before the statistical analysis of the KNHANES data was conducted, we examined the multicollinearity between variables based on the variance inflation factor (VIF). Additionally, we tested the linearity between the logit of the dependent variable and the independent variables using the Box–Tidwell test. The VIF values of all the variables, including BMI, WC, WHtR, TyG, TyG-BMI, TyG-WC, and TyG-WHtR, ranged from 1.00 to 1.33 for men and from 1.10 to 1.47 for women. Additionally, all the continuous independent variables met the linearity assumption because the *p* values of all the continuous independent variables ranged from 0.09 to 0.81 for men and from 0.40 to 0.99 for women according to the results of the Box–Tidwell test. We applied the Bonferroni correction to account for multiple comparisons arising from testing the seven indices such as BMI, WC, WHtR, TyG, TyG-BMI, TyG-WC, and TyG-WHtR. Accordingly, we set a more stringent threshold for statistical significance at a Bonferroni corrected *p* value < 0.007 (α = 0.05/7). Odds ratios (ORs) are shown with 95% confidence intervals (CIs), and a Bonferroni corrected *p* value < 0.05 was considered statistically significant.

## 3. Results

### 3.1. Basic Sociodemographic Characteristics of the Subjects

Table 1 presents the basic characteristics of the subjects who participated in this study. The prevalence of OA in the Korean population was 7.3% in men and 24.3% in women. The mean ages of the normal and OA groups were 58.73 ± 0.164 and 66.24 ± 0.53 years, respectively, for men (*p* < 0.001) and 57.9 ± 0.152 and 66.72 ± 0.253 years, respectively, for women (*p* < 0.001). In terms of sex differences, all the variables, including anthropometric indices, blood profiles, sociodemographic variables, and chronic diseases, exhibited significant differences between men and women (*p* < 0.001), except for the weekly working variable (*p* < 0.01). With respect to the difference between the normal and OA groups, in men, most variables exhibited significant differences except for BMI, glucose level, TyG-BMI, TyG-WC, marital status, weekly working status, and hypercholesterolemia. In women, all variables other than smoking exhibited significant differences between the normal and OA groups.

### 3.2. Associations of OA with the TyG Index and the TyG Index Combined with Obesity

Table 2 and Table 3 show the association of OA with the TyG index and TyG index combined with obesity among Korean men and women. Based on a Bonferroni corrected *p* value, the TyG index was not associated with OA in either the crude (OR = 0.89 [0.79–1.00], *p* = 0.315) or adjusted models (adj. OR = 0.98 [0.86–1.11], adj. *p* > 0.999) in men. Among the TyG indices combined with obesity, OA was not associated with TyG-BMI and TyG-WC in either the crude or adjusted models. TyG-WHtR was related to OA in the crude model (OR = 1.23 [1.09–1.38], *p* = 0.007), but this association disappeared in the adjusted model (adj. OR = 1.16 [1.03–1.31], adj. *p* = 0.103). The magnitudes of these associations were lower than those of the associations of OA with BMI, WC, and WHtR individually in both the crude and adjusted models.

In women, the TyG index was associated with OA (OR = 1.27 [1.19–1.35], *p* < 0.001); however, this association disappeared after adjustment to account for confounders (adj. OR = 1.08 [1.01–1.16], adj. *p* = 0.236). OA was strongly associated with TyG-BMI, TyG-WC, and TyG-WHtR in both the crude model (OR = 1.60 [1.50–1.70], *p* < 0.001; OR = 1.75 [1.64–1.87], *p* < 0.001; OR = 1.93 [1.80–2.07], *p* < 0.001) and the adjusted model (adj. OR = 1.44 [1.34–1.54], adj. *p* < 0.001; adj. OR = 1.40 [1.30–1.50], adj. *p* < 0.001; adj. OR = 1.39 [1.29–1.50], adj. *p* < 0.001). However, the magnitudes of these associations were slightly lower than those of the associations of OA with BMI, WC, and WHtR individually in both the crude and adjusted models.

In the interaction analysis between sex and TyG indices combined with obesity, the interaction effect was significant for all TyG indices combined with obesity (*p* for interaction < 0.001), except for the TyG index (*p* for interaction = 0.06) in the fully adjusted models, indicating that the associations between OA and TyG indices combined with obesity were stratified by sex. Overall, TyG was not associated with OA in both women and men. The magnitudes of the associations between all indices and OA were stronger in women than in men. TyG-BMI, TyG-WC, and TyG-WHtR were associated with OA in women, but not in men. In both men and women, the strengths of these associations were lower than those of the associations of OA with BMI, WC, and WHtR individually.

## 4. Discussion

In this study, we demonstrated that OA was not associated with the TyG index in both men and women. Additionally, TyG indices combined with obesity, such as the TyG-BMI, TyG-WC, and TyG-WHtR indices, were not associated with OA in men but strongly associated with OA in women. However, the strength of the associations between OA and TyG indices combined with obesity was lower than that of the association between OA and obesity indices individually.

Several studies have reported that the TyG index is associated with OA [7,12,13,15,16], whereas some studies have argued that the TyG index is only weakly associated or not associated with OA [11,17,18]. Cai et al. [19] examined the association between osteoarthritis and two insulin resistance indices (the homeostatic model assessment of insulin resistance (HOMA-IR) index and the TyG index) in a U.S. population. These authors reported that the TyG index was more strongly associated with OA than the HOMA-IR index, and the TyG-WHtR was the best predictor of OA among the TyG, TyG-BMI, TyG-WC, and TyG-WHtR indices. Zhang et al. [17] tested the association of arthritis (rheumatoid arthritis and osteoarthritis) with TyG–BMI, TyG–WC, and TyG–WHtR in Chinese and U.S. populations. These authors argued that the TyG–BMI and TyG–WHtR indices were positively associated with the incidence of arthritis in both populations and that these indices had greater diagnostic power than the TyG index. Additionally, Lan et al. [11] investigated the association of OA with TyG, TyG–BMI, TyG-WC, TyG–WHtR, the visceral adiposity index, and lipid accumulation products and explored the predictive power of these indices in the U.S. population. These authors reported that TyG-BMI, TyG-WHtR, TyG-WC, and LAP were positively related to the incidence of OA in an adjusted model and that the best predictor of OA was TyG-WHtR. However, TyG was not associated with the incidence of OA. Liu et al. [12] examined the association between new-onset arthritis and the TyG index through a prospective cohort study in a Chinese population and reported that the TyG index was an independent risk predictor of the start of new-onset arthritis in individuals aged 45 and above. Yan et al. [13] investigated the association between the TyG index and arthritis in a U.S. population and reported that the TyG index was positively associated with arthritis in older adults with normal weight and no diabetes. Additionally, Huang et al. [7] investigated whether elevated TyG values were associated with OA in a U.S. population and reported that elevated TyG values were linked to a high risk of OA. Kim et al. [18] tested the association of knee OA with HOMA-IR, TyG, TyG-BMI, and TyG-WC in the Korean population. These authors reported that HOMA-IR was not associated with knee OA, the TyG index was weakly associated with knee OA, and the TyG-BMI and TyG-WC indices were strongly associated with knee OA. Furthermore, Que et al. [15] examined the predictive power of estimated glucose disposal rate, TyG, TyG-BMI, TyG-WC, and TyG-WHtR for predicting OA. With respect to the prediction of OA, the estimated glucose disposal rate and the TyG, TyG-BMI, TyG-WC, and TyG-WHtR indices had areas under the receiver operating characteristic curve (AUCs) of 0.68, 0.58, 0.60, 0.62, and 0.64, respectively. Li et al. [16] investigated the potential mediating role of the TyG index in the association between sarcopenic obesity and OA in the U.S. population. These authors reported a significant association between the TyG index and elevated OA risk in subjects with sarcopenic obesity, and in this case, the TyG index had an AUC value of 0.572 for predicting OA risk. Our findings were not consistent with the results of most previous studies on this topic; namely, they indicated that the TyG index is strongly associated with OA [7,12,13,15,16]. However, our results were linked to the findings of previous studies in that they indicated that the TyG index was not associated or only weakly associated with OA [11,17,18]. With respect to TyG indices combined with obesity, both previous studies and this study reported that TyG indices combined with obesity indices such as TyG-BMI were associated with OA in both sexes [15,18,19] or predominantly in women. However, we assume that one of the reasons for these associations is that obesity indices are strongly associated with OA rather than TyG being associated with OA. For example, the results of previous studies [11,17,18,19] and this study revealed that TyG was weakly associated or not associated with OA; however, that TyG indices combined with obesity were strongly associated with OA because obesity indices were strongly associated with OA. Therefore, we speculate that obesity may explain these associations of OA with TyG-BMI, TyG-WC, and TyG-WHtR. In this study, obesity indices were strongly associated with OA, and TyG was weakly associated or not associated with OA. Similarly, Park et al. [21] investigated the association between obesity indices and knee OA using retrospective cohort data in the Korean population and reported that higher BMI and WC were associated with increased knee OA and that decreased obesity reduced the risk of knee OA. However, most previous studies have reported that the TyG value is strongly associated with OA [7,12,13,15,16]. Therefore, further research is needed to examine the interrelationships among obesity, TyG, and OA across various ethnic groups and countries.

This study had several limitations. First, as in other similar studies, the biological mechanism underlying the association between OA and TyG combined with obesity indices is difficult to explain because of the overlaps among various fields of lipids, obesity, and OA. Second, our findings cannot be generalized to other ethnic groups and countries because of the controversy over the association between TyG and OA to date. Third, because of the cross-sectional design, identifying cause–effect relationships between TyG values and OA as well as between TyG indices combined with obesity and OA is difficult. Fourth, the KNHANES dataset used in this study only provides doctor-diagnosed OA and self-reported current OA symptoms based on questionnaires. Therefore, our findings should be interpreted with caution and regarded as preliminary evidence. Fifth, our results were derived from the Korean population, and the criteria for obesity based on BMI differ across ethnic groups. Therefore, further research is needed to examine the associations between TyG index values and OA in other ethnic groups and countries to ensure external validity, as well as to clarify the causal relationships and underlying biological mechanisms. However, this study has several strengths. The statistical findings concerning the relationship of OA with TyG values and TyG values combined with obesity are powerful because of the use in this research of KNHANES data supported by a national representative sample of the Korean population.

## 5. Conclusions

In this study, we examined the association of OA with the TyG index and TyG indices combined with obesity in the Korean population. Our findings revealed that OA was not associated with the TyG index in both men and women. Additionally, TyG indices combined with obesity were not associated with OA in men but strongly associated with OA in women. Additionally, combining TyG values and obesity indices to identify OA is not appropriate because obesity indices individually were better predictors than combined indices. Our findings were derived from a single cross-sectional study in the Korean population. So, the generalization of our results is limited. Therefore, our findings should be interpreted with caution and considered as preliminary evidence that requires further investigation.

## Figures and Tables

**Table 1:** Sociodemographic characteristics of the subjects.

Variable		Men			Women		
		Normal	OA	*p* Value	Normal	OA	*p* Value
Subjects (*n*)		6098 (92.7)	477 (7.3)		6471 (75.7)	2082 (24.3)	
Age (years) ***		58.73 ± 0.164	66.24 ± 0.53	<0.001	57.9 ± 0.152	66.72 ± 0.253	<0.001
Height (cm) ***		168.9 ± 0.101	166.7 ± 0.325	<0.001	156.2 ± 0.091	153.8 ± 0.154	<0.001
Weight (kg) ***		69.52 ± 0.158	68.1 ± 0.546	0.012	57.8 ± 0.124	59.44 ± 0.242	<0.001
WC (cm) ***		86.89 ± 0.127	88.64 ± 0.473	<0.001	80.34 ± 0.143	85.29 ± 0.244	<0.001
WHtR (ratio) ***		0.515 ± 0.001	0.532 ± 0.003	<0.001	0.515 ± 0.001	0.555 ± 0.002	<0.001
BMI (ratio) ***		24.32 ± 0.045	24.46 ± 0.166	0.396	23.67 ± 0.049	25.11 ± 0.086	<0.001
SBP (mmHg) ***		122.7 ± 0.256	126 ± 0.864	<0.001	120.3 ± 0.267	126.6 ± 0.438	<0.001
DBP (mmHg) ***		78.44 ± 0.161	75.63 ± 0.565	<0.001	75.25 ± 0.147	74.65 ± 0.255	0.035
Triglyceride (mg/dL) ***		162.5 ± 2.378	145.6 ± 5.288	0.003	123 ± 1.139	136.8 ± 2.505	<0.001
Glucose (mg/dL) ***		107 ± 0.389	108.6 ± 1.609	0.312	100.4 ± 0.333	104.1 ± 0.552	<0.001
TyG (ratio) ***		8.857 ± 0.01	8.786 ± 0.034	0.04	8.572 ± 0.009	8.705 ± 0.016	<0.001
TyG-BMI (ratio) ***		215.9 ± 0.519	215.2 ± 1.767	0.696	203.5 ± 0.544	219.1 ± 0.927	<0.001
TyG-WC (ratio) ***		771.4 ± 1.608	780 ± 5.601	0.134	690.5 ± 1.652	744 ± 2.841	<0.001
TyG-WHtR (ratio) ***		4.568 ± 0.009	4.679 ± 0.033	0.001	4.427 ± 0.011	4.846 ± 0.019	<0.001
Education level ***	Middle school or below	30.6 (0.82)	57.2 (2.8)	<0.001	39.8 (0.85)	73.4 (1.23)	<0.001
	High school	33 (0.75)	28.5 (2.53)		37.5 (0.77)	19.2 (1.03)	
	University or above	36.4 (0.94)	14.2 (2.06)		22.7 (0.75)	7.4 (0.68)	
Household income (quartile) ***	Low	16.6 (0.6)	38.6 (2.7)	<0.001	19.8 (0.7)	40.6 (1.32)	<0.001
	Middle-low	24.2 (0.7)	28.7 (2.6)		24.3 (0.7)	24.9 (1.04)	
	Middle-high	26.9 (0.7)	19.7 (2.2)		25.3 (0.7)	19.8 (1.03)	
	High	32.3 (0.9)	12.9 (1.9)		30.6 (0.8)	14.7 (1.01)	
Marital status ***	Married	87.4 (0.56)	83.7 (2.3)	0.076	76.1 (0.7)	59.4 (1.32)	<0.001
	Single	12.6 (0.56)	16.3 (2.3)		23.9 (0.7)	40.6 (1.32)	
Smoking ***	No	67.4 (0.8)	78.8 (2.4)	<0.001	95.9 (0.31)	96.9 (0.5)	0.103
	Yes	32.6 (0.8)	21.2 (2.4)		4.1 (0.31)	3.1 (0.5)	
Alcohol consumption ***	No	19.4 (0.59)	26.8 (2.3)	0.001	39.6 (0.74)	52.9 (1.3)	<0.001
	Yes	80.6 (0.59)	73.2 (2.3)		60.4 (0.74)	47.1 (1.3)	
Weekly walking (4+ h) **	No	62.9 (0.77)	64.5 (2.52)	0.552	64.2 (0.72)	72 (1.15)	<0.001
	Yes	37.1 (0.77)	35.5 (2.52)		35.8 (0.72)	28 (1.15)	
Menopause	No				29.9 (0.7)	7.9 (0.72)	<0.001
	Yes				70.1 (0.7)	92.1 (0.72)	
Hypertension ***	No	56.9 (0.76)	45.4 (2.8)	<0.001	65.6 (0.7)	44.4 (1.3)	<0.001
	Yes	43.1 (0.76)	54.6 (2.8)		34.4 (0.7)	55.6 (1.3)	
Diabetes ***	No	81.6 (0.56)	74.7 (2.4)	0.002	87.6 (0.5)	79.3 (1)	<0.001
	Yes	18.4 (0.56)	25.3 (2.4)		12.4 (0.5)	20.7 (1)	
Hypercholesterolemia ***	No	77.3 (0.63)	74 (2.31)	0.152	69.4 (0.65)	57.3 (1.2)	<0.001
	Yes	22.7 (0.63)	26 (2.31)		30.6 (0.65)	42.7 (1.2)	
Hypertriglyceridemia ***	No	77.3 (0.65)	83 (1.95)	0.011	88.6 (0.47)	85.2 (0.96)	<0.001
	Yes	22.7 (0.65)	17 (1.95)		11.4 (0.47)	14.8 (0.96)	

*p* values were obtained using Rao–Scott chi-square tests for categorical variables and a general linear model for continuous variables between the normal group and OA group. The values ** *p* < 0.01, *** *p* < 0.001) represent the *p* values for sex differences. Continuous variables are presented as the means ± standard errors (SEs), and categorical variables are presented as percentages (SEs). Abbreviations: OA: osteoarthritis, BMI: body mass index, WC: waist circumference, WHtR: waist-to-height ratio, SBP: systolic blood pressure, DBP: diastolic blood pressure, TyG: triglyceride–glucose value.

**Table 2:** Associations of OA with the TyG index and TyG indices combined with obesity in Korean men.

Variable	Crude Model		Adjusted Model	
	OR (95% CI)	*p* Value	Adj. OR (95% CI)	Adj. *p* Value
BMI	1.07 (0.92–1.25)	>0.999	1.27 (1.08–1.49)	0.029
WC	1.41 (1.17–1.70)	0.002	1.40 (1.17–1.69)	0.002
WHtR	1.50 (1.32–1.70)	<0.001	1.27 (1.12–1.45)	0.002
TyG	0.89 (0.79–1.00)	0.315	0.98 (0.86–1.11)	>0.999
TyG-BMI	0.98 (0.87–1.10)	>0.999	1.14 (1.01–1.30)	0.292
TyG-WC	1.10 (0.97–1.24)	0.915	1.17 (1.03–1.32)	0.123
TyG-WHtR	1.23 (1.09–1.38)	0.007	1.16 (1.03–1.31)	0.103

OR (95% CI) and *p* values in the crude and adjusted models were obtained using complex sample binary logistic regression. A Bonferroni corrected *p* value < 0.05 was considered statistically significant. Crude model: not adjusted. Adjusted model: adjusted to account for age, education, alcohol consumption, household income, smoking status, marital status, and weekly walking. Abbreviations: TyG: triglyceride–glucose index, BMI: body mass index, WC: waist circumference, WHtR: waist-to-height ratio, CI: confidence interval, OR: odds ratio, Adj.: adjusted.

**Table 3:** Associations of OA with the TyG index and TyG indices combined with obesity among Korean women.

Variable	Crude Model		Adjusted Model	
	OR (95% CI)	*p* Value	Adj. OR (95% CI)	Adj. *p* Value
BMI	1.69 (1.58–1.81)	<0.001	1.58 (1.46–1.70)	<0.001
WC	2.13 (1.96–2.31)	<0.001	1.62 (1.48–1.77)	<0.001
WHtR	2.10 (1.95–2.25)	<0.001	1.48 (1.37–1.60)	<0.001
TyG	1.27 (1.19–1.35)	<0.001	1.08 (1.01–1.16)	0.236
TyG-BMI	1.60 (1.50–1.70)	<0.001	1.44 (1.34–1.54)	<0.001
TyG-WC	1.75 (1.64–1.87)	<0.001	1.40 (1.30–1.50)	<0.001
TyG-WHtR	1.93 (1.80–2.07)	<0.001	1.39 (1.29–1.50)	<0.001

OR (95% CI) and *p* values in the crude and adjusted models were obtained using complex sample binary logistic regression. A Bonferroni corrected *p* value < 0.05 was considered statistically significant. Crude model: not adjusted. Adjusted model: adjusted to account for age, education, alcohol consumption, household income, smoking status, marital status, weekly walking, and menopause. Abbreviations: TyG: triglyceride–glucose index, BMI: body mass index, WC: waist circumference, WHtR: waist-to-height ratio, CI: confidence interval, OR: odds ratio, Adj.: adjusted.

## Data Availability

All the data used in the present study are available from the Korea National Health and Nutrition Examination Survey (KNHANES) conducted by the Korea Centers for Disease Control and Prevention (KCDC). The data can be accessed freely (https://knhanes.kdca.go.kr/knhanes/eng/main.do and https://knhanes.kdca.go.kr/knhanes/main.do) (accessed on 1 September 2025).
